# Assessment of serum symmetric dimethylarginine and creatinine concentrations in hyperthyroid cats before and after a fixed dose of orally administered radioiodine

**DOI:** 10.1111/jvim.15831

**Published:** 2020-06-07

**Authors:** Lucia Yu, Lauren Lacorcia, Sue Finch, Thurid Johnstone

**Affiliations:** ^1^ Translational Research and Small Animal Clinical Trial Study Group, Faculty of Veterinary and Agricultural Sciences The University of Melbourne Victoria Australia; ^2^ Statistical Consulting Centre and Melbourne Statistical Consulting Centre The University of Melbourne Victoria Australia

**Keywords:** azotemia, chronic kidney disease, CKD, feline, hyperthyroidism, I‐131

## Abstract

**Background:**

Serum symmetric dimethylarginine (SDMA) is a sensitive renal biomarker for detecting early chronic kidney disease (CKD) in nonhyperthyroid cats, but knowledge regarding its performance in hyperthyroid cats remains limited.

**Objectives:**

To determine the relationship between serum SDMA, creatinine and total thyroxine (TT4) concentrations in hyperthyroid cats before (T0) and 3 months after (T1) receiving a PO fixed dose of radioiodine.

**Animals:**

Eighty client‐owned hyperthyroid cats.

**Methods:**

Prospective cohort study. Serum TT4, and SDMA, creatinine concentrations, and urine specific gravity were measured at T0 and T1. Nonparametric tests were used to determine the relationship among SDMA, and creatinine and TT4 concentrations. Agreement between SDMA and creatinine regarding CKD staging at both time points was assessed using Goodman and Kruskal's gamma statistic.

**Results:**

Mean serum SDMA concentration increased after treatment of hyperthyroidism. However, 21 of 75 cats experienced a decrease in SDMA between T0 and T1, whereas creatinine decreased in only 2 cats. A moderate correlation between SDMA and creatinine was seen at T1 (*r* = 0.53; *P <* .001) but not at T0 (*r* = 0.13; *P =* .25). Where assessable at T1, poor agreement was observed between SDMA and creatinine and CKD stage (Goodman and Kruskal's gamma 0.20; *P =* .29).

**Conclusions and clinical importance:**

Discordant outcomes between SDMA and creatinine after radioiodine treatment in cats with hyperthyroidism suggest extrarenal factors may interfere with the reliability of SDMA to adequately reflect renal function. As a result, SDMA should not be interpreted in isolation in hyperthyroid cats treated with radioiodine.

**Abbreviations**
BCS: body condition score
CKD: chronic kidney disease
GFR: glomerular filtration rate
IRIS: International Renal Interest Society
MCS: muscle condition score
SDMA: symmetric dimethylarginine
TT4: total thyroxine
USG: urine specific gravity

## 1 INTRODUCTION

Hyperthyroidism and chronic kidney disease (CKD) are common in older cats. Previous studies indicate that 10% to 49% of cats with hyperthyroidism have concurrent renal azotemia diagnosed before or after treatment.^1^, ^2^, ^3^, ^4^, ^5^, ^6^ Chronic kidney disease has implications for the clinical decision‐making process regarding optimal treatment options for hyperthyroidism and may decrease the lifespan of affected cats. (Wakeling J, Rob C, Elliot J, et al. Survival of hyperthyroid in cats is not affected by post‐treatment azotaemia. Proceedings of the 17th ECVIM‐CA Congress, JVIM 2006; 20:1523.)^7^ The increase in thyroid hormone production in hyperthyroidism increases renal blood flow and glomerular capillary hydrostatic pressure, thereby increasing glomerular filtration rate (GFR). Indirect measures of GFR such as serum creatinine and urea nitrogen concentrations are notoriously inaccurate at detecting CKD in hyperthyroid cats. Their inaccuracy is largely attributable to the effects of extrarenal factors such as muscle mass, as well as daily variability in exogenous and endogenous protein loads.^8^, ^9^, ^10^, ^11^

Pretreatment GFR has been proposed as a potential predictor of post‐treatment azotemia.^5^, ^12^, ^13^ However, sensitive and specific cut‐offs have proved difficult to establish.^5^, ^12^ Although single sample plasma clearance methods have been validated in cats, these are yet to be assessed in hyperthyroid cats and currently are not widely available in the clinical setting.^14^, ^15^

Symmetric dimethylarginine (SDMA) is an indirect biomarker of GFR that is unaffected by muscle mass and reportedly detects CKD with higher sensitivity than creatinine.^11^, ^16^ Recently, SDMA has been incorporated as an adjunctive variable in the International Renal Interest Society (IRIS) guidelines for CKD staging and management.^17^ However, the influence of hyperthyroidism on SDMA in cats is yet to be explored in detail.

It has been reported that increases in serum SDMA concentration in nonazotemic hyperthyroid cats before treatment are relatively insensitive, but specific for predicting azotemia after treatment.^18^ However, other studies have failed to establish a significant correlations among SDMA, GFR and total thyroxine (TT4) and have suggested that the serum SDMA concentration may not always differ between hyperthyroid and healthy cats. (Corsini A, Crosara, S, Carotenuto G, et al. Symmetric Dimethylarginine (SDMA) in Hyperthyroid Cats. Proceedings of the 27th ECVIM‐CA Congress in JVIM 2017;32:588. Williams TL. Serum symmetric dimethylarginine concentrations in hyperthyroid cats with and without azotemic chronic kidney disease. Proceedings of the 2017 ACVIM Forum in JVIM 2017;31:1274.)^19^ Further exploration of the relationships among SDMA, creatinine and TT4 in another population of hyperthyroid cats is warranted.

Our objectives were to prospectively measure serum SDMA concentrations in hyperthyroid cats receiving a fixed PO dose of radioiodine before and after treatment and to assess serum SDMA concentration in relation to serum creatinine and TT4 concentrations at both time points. It was hypothesized that serum SDMA concentration would increase significantly as hyperthyroidism resolves. Serum SDMA and creatinine concentrations were expected to be significantly correlated only after hyperthyroidism resolved, leaving fewer extrarenal factors to affect serum creatinine concentration. After radioiodine treatment, where renal dysfunction was suspected based on serum creatinine concentration and concurrent suboptimal urine specific gravity (USG), serum SDMA concentration was hypothesized to be concurrently increased.

## 2 MATERIALS AND METHODS

### 2.1 Sample population

This study was a prospective cohort study of client‐owned cats with a confirmed diagnosis of hyperthyroidism and deemed appropriate for treatment with a fixed (approximately 138Mbq; 3.7 mCi) PO dose of radioiodine at the University of Melbourne's U‐Vet Werribee Animal Hospital. The enrollment period was from January 2017 to September 2018. All procedures included in the study were approved by the Animal Ethics Committee of the University of Melbourne (AEC application ID 1613858) and all owners gave written consent to have their cats included in the study.

To be eligible for inclusion in the study, cats were required to meet the inclusion and exclusion criteria of a previously established standard radioiodine treatment protocol.^20^

Cats were eligible for treatment with fixed dose radioiodine after a definitive diagnosis of hyperthyroidism was made based on presence of clinical signs consistent with hyperthyroidism (eg, weight loss despite polyphagia, polydipsia and polyuria, unkempt coat)^1^ and serum TT4 concentration above the upper limit of the reference interval.

### 2.2 Data and sample collection

Age, sex and neuter status were recorded for each cat. At initial presentation (T0) and at least 3 months (T1) after radioiodine treatment, historical information collected included any clinical signs attributable to hyperthyroidism.^21^ All cats were examined by the primary clinician; either a resident or board‐certified (ACVIM or ECVIM‐CA) specialist in small animal internal medicine. The physical examination included blood pressure measurement using Doppler sphygmomanometry,^22^ body weight, body condition score (BCS), and muscle condition score (MSC). The BCS was assigned by a validated 9‐point system,^23^ whereas the MCS was assigned by a 4‐point system.^24^

Blood and urine (voided or cystocentesis collection) were sampled at T0 and T1 to measure clinicopathological variables as outlined below. Where possible, samples were collected from unsedated cats, but fractious cats had sample collection performed under IM sedation. Samples that were collected after sedation were flagged, and the type and amount of sedation used were recorded.

### 2.3 Clinicopathological assays

Blood collected into serum tubes was centrifuged within 1 hour of collection and serum separated for standard biochemistry analysis within 2 hours of collection at the clinical pathology laboratory of the U‐Vet Animal Hospital by standardized methods. Laboratory technicians manually read the packed cell volume (PCV) after centrifugation of blood in hematocrit tubes, and determined the total protein concentration of the serum and USG with a handheld, calibrated refractometer.

The creatinine assay was a standard kinetic colorimetric assay based on the Jaffé method, (CREJ2, Roche Diagnostics Ltd, Switzerland) and the urea assay was a standard kinetic colorimetric assay based on urease and glutamate dehydrogenase. (UREAL, Roche Diagnostics Ltd, Switzerland).

At T0, the remaining serum was separated in 0.25 mL aliquots and stored at −80°C until the T1 sample was acquired. At this time, the T0 samples were thawed to allow paired analysis of T0 and T1 samples for TT4 and SDMA concentrations. Serum TT4 concentration was measured using a solid‐phase competitive chemiluminescence immunoassay (Immulite 1000 XPI, Siemens, Victoria, Australia), previously validated for use in cats.^25^ The reference interval for the TT4 assay was 7.4 to 35.0 μg/L (9.5‐48.0 nmol/L), as validated by method comparison to the solid‐phase competitive chemiluminescence immunoassay.^25^, ^26^ The lower limit of the range of linearity of the assay was 5.0 μg/L (6.4 nmol/L), whereas the upper limit of the range of linearity of the assay was 149.9 μg/L (193.0 nmol/L). Serum SDMA concentration was measured at IDEXX laboratories (Victoria, Australia) using a high‐throughput biochemistry analyzer (Beckman and Coulter AU680, Beckman and Coulter Pty Ltd, Victoria, Australia) validated as previously described. (Prusevich P, Patch D, Obare E, et al. Validation of a novel high throughput immunoassay for the quantification of symmetric dimethylarginine (SDMA). Proceedings of the American Association for Clinical Chemistry Annual Meeting and Clinical Lab Expo., Georgia, Atlanta 2015; S135. Patch D, Obare E, Prusevich P, et al. High throughput immunoassay for kidney function biomarker symmetric dimethylarginine (SDMA). Proceedings of the American Association for Clinical Chemistry Annual Meeting and Clinical Lab Expo., Georgia, Atlanta 2015; S135.) The IDEXX laboratory was otherwise not involved in the study and was blinded to all other study data. Similarly, the hospital clinical pathology laboratory was otherwise not involved in the study and was blinded to all other study data.

### 2.4 Defining kidney dysfunction

Renal azotemia was defined as a serum creatinine concentration of ≥1.6 mg/dL (≥140 μmol/L) or a serum SDMA concentration of ≥18 μg/dL with concurrent USG <1.035.^27^, ^28^ The severity of kidney dysfunction then was stratified separately into CKD stages based on serum creatinine and SDMA concentrations according to IRIS CKD staging guidelines.^27^ Where urine concentration was ≥1.035, prerenal azotemia was defined as a concurrent serum creatinine concentration ≥1.6 mg/dL (≥140 μmol/L) or serum SDMA concentration ≥14 μg/dL. Possible IRIS stage 1 CKD was defined by documenting a single USG <1.035 with a concurrent serum creatinine concentration <1.6 mg/dL (<140 μmol/L) or serum SDMA concentration <18 μg/dL.^27^

### 2.5 Statistical analysis

All statistical analyses were performed by Minitab version 18 (Minitab LLC, State College, PA, United States). Sample size calculations were performed by the McNemar test to determine the number of cats needed to identify potential differences between SDMA and creatinine in detecting early CKD post‐radioiodine treatment. An effect size of 15% was used, with power of 80% and alpha set to 0.05% in the Asia Pacific region between 2015 and 2016 (IDEXX laboratories, New South Wales, Australia).

Continuous variables were assessed for normality by visual inspection of graphical plots. Results were reported as median with interquartile ranges (IQR) where data was not consistent with a normal distribution, and as mean and SD where data appeared normally distributed. Paired *t* tests were used for comparison of the means of continuous variables (eg, serum urea, creatinine, SDMA and TT4 concentrations and USG) at T0 and T1. The Wilcoxon signed rank test was used to compare BCS and MCS at T0 and T1. Correlations between TT4 and SDMA, TT4 and creatinine and SDMA and creatinine at T0 and T1 were assessed, respectively, by the Spearman rank correlation coefficient. All TT4 concentrations <5.0 μg/L (<6.4 nmol/L) were assigned the value of 5.0 μg/L when determining the Spearman rank correlation coefficient. Similarly, TT4 concentrations >145.0 μg/L (>193.0 nmol/L) were assigned the value of 145.0 μg/L.

Outliers were defined as being ≥3 SD from the mean. When outliers were detected, sensitivity analyses were conducted to investigate the influence of the outliers on the relationships between the variables. The Goodman and Kruskal's gamma statistic was used as a measure of concordance of categorization of IRIS CKD staging between creatinine and SDMA. Multivariable analysis by a general linear model explored the influence of extrarenal variables such as BCS and MCS on SDMA and creatinine. For all analyses, statistical significance was defined as *P* < .05 and all statistical tests were 2‐tailed.

## 3 RESULTS

### 3.1 Sample characteristics

Based on sample size calculations, data from at least 76 cats were required for the study to have sufficient power to detect a difference between SDMA and creatinine for detection of CKD at T1. A drop‐out rate up to 40% was expected, and thus in the period of inclusion, 108 cats treated with fixed dose radioiodine were enrolled with owner consent. The mean calibrated dose of PO radioiodine used was 3.6 mCi (131.6 MBq; SD, 0.1 mCi or 5.0 MBq). Twenty‐eight (26%) cats did not complete the study or were excluded: owners of 16 cats declined follow‐up examinations at the study institution because of travel distance, owners of 11 cats could not be contacted and 1 cat was diagnosed with large cell gastrointestinal lymphoma before T1. Eighty (74%) cats returned for their T1 evaluation and were included in the study. The median duration from treatment to re‐evaluation was 108 days (IQR, 99‐134 days).

The 80 included cats consisted of 70 domestic shorthair cats, 5 domestic medium or longhair cats, 2 Ragdolls and 1 each of Tonkinese and Burmese breeds. There were 47 (59%) female neutered and 33 (41%) male neutered cats. The median age was 12.9 years (IQR, 10.6‐15.1). Mean body weight, BCS and MCS increased, and mean systolic blood pressure decreased significantly after treatment (Supporting Information Table **S1**). At T0, 17 cats had serum TT4 concentrations >15.0 μg/dL (>193.0 nmol/L). At T1 (after radioiodine treatment), 2 (2.5%) cats had persistent hyperthyroidism based on serum TT4 concentrations above the upper limit of the reference interval. Sixty‐six (82.5%) cats had serum TT4 concentrations within the reference interval and 12 (15%) cats had serum TT4 concentrations below the lower limit of the reference interval. Of the latter cats, 7 had serum TT4 concentrations <0.5 μg/dL (<6.4 nmol/L). At T0, 26 (33%) cats received IM sedation before urine and blood sampling, whereas 3 cats received IM sedation at T1.

### 3.2 Renal clinicopathological parameters before and after radioiodine treatment

Serum SDMA concentrations were not available on 6 occasions (3 each at T0 and T1) because of leakage of sample during transit to the IDEXX laboratory. The USG could not be measured in 4 cats at T1 because of empty urinary bladders and inability to obtain a sample.

**FIGURE 1:**
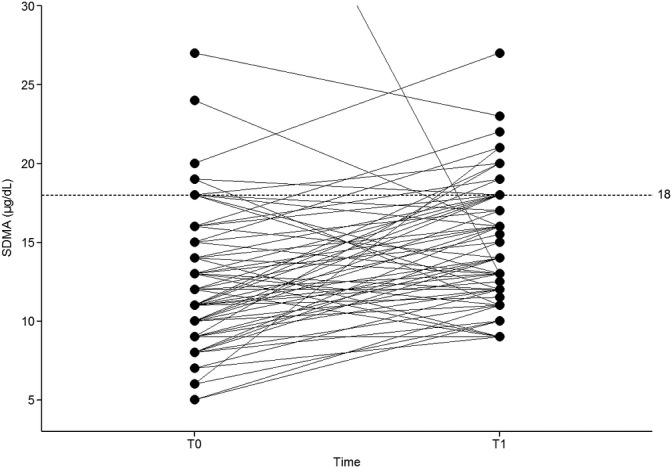
Serum symmetric dimethylearginine (SDMA) of 75 cats before and after radioiodine treatment *Note:* T0, before treatment; T1, after treatment. The dotted line represents the upper reference limit for SDMA. To optimize the scale of this graph, 2 cats with outlier values were excluded. The SDMA concentration of the first outlier was 49.4 μg/dL (T0) and 13.0 μg/dL (T1), whereas the SDMA concentration of the second outlier was 47.0 μg/dL (T0) and 118.0 μg/dL (T1)

Serum SDMA concentration increased in 52 of 75 (69%) cats, decreased in 21 (28%) cats and was unchanged in 2 (3%) cats from T0 to T1 (Figure 1). Of the 21 cats with serum SDMA concentrations decreased between T0 and T1, 18 cats had a concurrent increase in total protein concentration. Sixteen of the cats were euthyroid and 5 had serum TT4 concentrations below the lower limit of the reference interval. The 2 cats with unchanged SDMA concentration at T0 and T1 were both euthyroid at T1.

Serum creatinine concentration increased in 78 of 80 (96%) cats, decreased in 2 (3%) cats and was unchanged in1 (1%) cat from T0 to T1 (Supporting Information Figure S1). Those cats with decreased or static serum creatinine concentrations from T0 to T1 all were euthyroid at T1. Urine specific gravity decreased in 49 of 76 (64%) cats, increased in 19 (25%) cats and was unchanged in 8 (11%) cats from T0 from T1 (Supporting Information Figure S2). At T0, 19 of 80 (24%) cats had USG <1.035, of which 1 (1%) was considered to have renal azotemia. At T1, 37 of 76 (49%) cats had USG <1.035, of which 26 (34%) were considered to have renal azotemia.

In the total sample population of 80 cats, PCV decreased and total protein concentration increased significantly at T1 compared to T0. A significant increase in the means of serum creatinine, urea and SDMA occurred, whereas mean serum TT4 and USG decreased significantly in the total sample at T1 (Table 1). Similarly, when only cats with serum TT4 concentrations within the reference interval (ie, those considered euthyroid) at T1 were considered (n = 66 cats), a significant increase in mean serum creatinine, urea and SDMA concentrations occurred from T0 to T1, whereas the mean USG decreased significantly from T0 to T1 (Supporting Information Table S2). In this group of cats, 32 of 62 (51%) had USG < 1.035 at T1, of which 21 cats (34% of the subgroup) had renal azotemia based on the serum creatinine concentration and USG. Ten of these 21 azotemic cats had been nonazotemic with USG < 1.035 at T0. Five cats had USG < 1.035 at both time points but remained nonazotemic. Only 2 cats with USG <1.035 at T0 had an increase in USG to ≥1.035 at T1, neither of these cats had renal azotemia based on the serum creatinine concentration at T0.

**TABLE 1:** Clinicopathologic data of 80 hyperthyroid cats before (T0) and after (T1) radioiodine treatment

	T0		T1		Estimated difference; T1‐T0 (95% confidence interval)	*P*‐value^a^
	N	Mean (SD)	N	Mean (SD)		
PCV (%)	80	36.4 (4.7)	80	33.7 (4.1)	−2.7 (−3.7, −1.8)	<.001
TP (g/dL)	80	68.9 (5.5)	80	74.6 (5.3)	5.8 (7.1, 4.5)	<.001
Serum TT4 (μg/dL)	80	10.5 (4.4)	80	1.6 (1.4)	−8.9 (−9.9, −7.8)	<.001
Serum creatinine (mg/dL)	80	0.9 (0.3)	80	1.6 (0.4)	0.7 (0.6, 0.8)	<.001
Serum urea (mg/dL)	80	23.5 (7.0)	80	29.4 (8.4)	5.9 (4.2, 7.3)	<.001
Serum SDMA (μg/dL)	75	13.1 (7.2)	75	15.9 (12.5)	2.8 (0.5, 5.1)	.02
Urine specific gravity	76	1.041 (0.010)	76	1.034 (0.014)	−0.007 (−0.010, −0.004)	<.001

Abbreviations: T0, before treatment; T1, after treatment; TP, total protein; TT4, total thyroxine; SDMA, symmetric dimethylarginine.

^a^ Paired *t* test.

In the 12 cats with a serum TT4 concentration below the lower limit of the reference interval at T1, mean serum SDMA concentrations were not significantly different between T0 (16.0 μg/dL; SD, 13.10) and T1 (12.3 μg/dL; SD, 3.9; *P* = .36. [paired *t* test]). However, mean serum concentrations for creatinine and urea increased whereas mean USG decreased significantly from T0 to T1 (Supporting Information Table S3). Five (41.6%) of 12 cats were considered to have renal azotemia based on the serum creatinine concentration and USG at T1, in which USG < 1.035 had been present at T0 in 2 of 5 cats.

In the 2 cats that remained hyperthyroid at T1, both cats had USG ≥1.035, with normal serum creatinine concentrations at T0 and T1. The serum SDMA concentrations were within the normal reference interval for both cats at T0. At T1, the serum SDMA concentration remained normal 1 cat, and increased slightly above the reference interval in the other.

### 3.3 Categorization of renal dysfunction by SDMA or creatinine before and after radioiodine treatment

The categorization of IRIS stages at T0 based on both serum creatinine and SDMA concentration, is summarized in Table 2. Seventy‐seven (96%) of 80 cats had complete renal clinicopathological information including serum SDMA and creatinine concentrations and USG. Of the 3 cats with missing serum SDMA concentration, 1 had a USG ≥1.035 and 2 had USG <1.035; these were recorded as missing values in Table 2. The latter cats were classified as having IRIS stage 2 CKD based on serum creatinine concentration. Serum SDMA and creatinine concentrations agreed on IRIS CKD staging in 13 of 17 (76%) cats; 12 with possible IRIS stage 1 CKD and 1 with stage 2 CKD. Based on serum SDMA concentrations, 4 cats were classified as having stage 2 CKD or higher, whereas only 1 cat was classified as having stage 2 CKD and none had higher stages of CKD based on serum creatinine concentration. The number of cats with USG < 1.035 was not large enough to perform valid statistical testing to assess concordance of categorization of IRIS staging between serum SDMA and creatinine concentration for this subgroup. Only 1 of 26 cats given IM sedation before blood and urine sampling had discordant staging between SDMA and creatinine at T0. Thus, the effect of sedation on IRIS CKD staging between the variables was not further assessed statistically.

**TABLE 2:** International Renal Interest Society chronic kidney disease staging before radioiodine treatment with serum creatinine and SDMA concentrations

IRIS CKD stage		Based on serum creatinine			
		0	1	2	Total
Based on serum SDMA	0	**60**	…	…	60
	1	…	**12**	0	12
	2	…	2	**1**	3
	4	…	2	0	2
	Missing	1	2	0	3
	Total	61	18	1	80

*Note:* International Renal Interest Society CKD staging (0–4) according to the creatinine (columns) and SDMA (rows) concentrations. The allocation of stage 0 is given to cats with adequate urine concentration (urine specific gravity ≥1.035) and deemed to have adequate renal function. The allocation of possible stage 1 is given to cats with urine specific gravity <1.035, but with creatinine <140 μmol/L and/or SDMA <18 μg/dL. Bolded numbers indicate agreement between serum creatinine and SDMA.

Abbreviations: CKD, chronic kidney disease; SDMA, symmetric dimethylarginine.

The categorization of IRIS stage based on serum creatinine or SDMA concentration, respectively, at T1 is summarized in Table 3. Seventy‐four (93%) of 80 cats had complete renal clinicopathological information including serum SDMA and creatinine concentrations and USG. As mentioned previously, 3 cats had no serum SDMA concentration measured and 4 cats had no USG measured; these were recorded as missing values in Table 3. Of the cats with missing SDMA, 1 had USG ≥1.035, 1 had USG <1.035 and 1 had no USG measured. Of the cats with complete data, 38 of 74 (51%) had USG ≥1.035. Serum SDMA and creatinine concentrations agreed with IRIS CKD staging in 17 of 36 (47%) cats, 8 with possible stage 1 CKD and 9 with stage 2 CKD. Based on serum SDMA concentration, 13 cats were classified as having stage 2 CKD or higher, whereas based on serum creatinine concentration, 26 cats were classified as having stage 2 CKD and none with higher stages of CKD (Table 3). None of the cats with discordant staging results received sedation at T1.

**TABLE 3:** International Renal Interest Society chronic kidney disease staging after radioiodine treatment with serum creatinine and symmetric dimethylarginine concentrations

IRIS CKD stage		Based on serum creatinine				
		0	1	2	Missing	Total
Based on SDMA	0	**38**	…	…	…	38
	1	…	**8**	15	0	23
	2	…	2	**9**	0	11
	3	…	0	1	0	1
	4	…	1	0	0	1
	Missing	1	0	1	4	6
	Total	39	11	26	4	80

*Note:* International Renal Interest Society CKD staging (0–4) according to the creatinine (columns) and SDMA (rows) concentrations. The allocation of stage 0 is given to cats with adequate urine concentration (urine specific gravity ≥1.035) and deemed to have adequate renal function. The allocation of possible stage 1 is given to cats with urine specific gravity <1.035, but with creatinine <140 μmol/L and/or SDMA <18 μg/dL. Bolded numbers indicate agreement between serum creatinine and SDMA.

Abbreviations: CKD, chronic kidney disease; SDMA, symmetric dimethylarginine.

For all cats at T1, poor agreement of IRIS CKD staging was found when comparing classification by creatinine with SDMA; the Goodman and Kruskal's gamma was 0.20 and did not achieve statistical significance (*P =* .29).

### 3.4 Correlation between serum SDMA and creatinine before and after radioiodine treatment

Correlation between SDMA and creatinine at T0 and T1 was assessed in 77 cats. No significant correlation was found between serum SDMA and creatinine concentrations at T0 (*r* = 0.13; 95% CI, −0.10, 0.35; *P* = .25) when the entire sample was included. Two cats were identified as outliers based on their SDMA concentrations at T0, which were approximately 5 SDs above the mean SDMA concentration. Both cats were classified as having possible IRIS stage 1 CKD based on their serum creatinine concentrations and USG at T0. When these outliers were removed, the correlation between SDMA and creatinine at T0 changed minimally (*r* = 0.10; 95% CI, −0.13, 0.32; *P* = .38).

**FIGURE 2:**
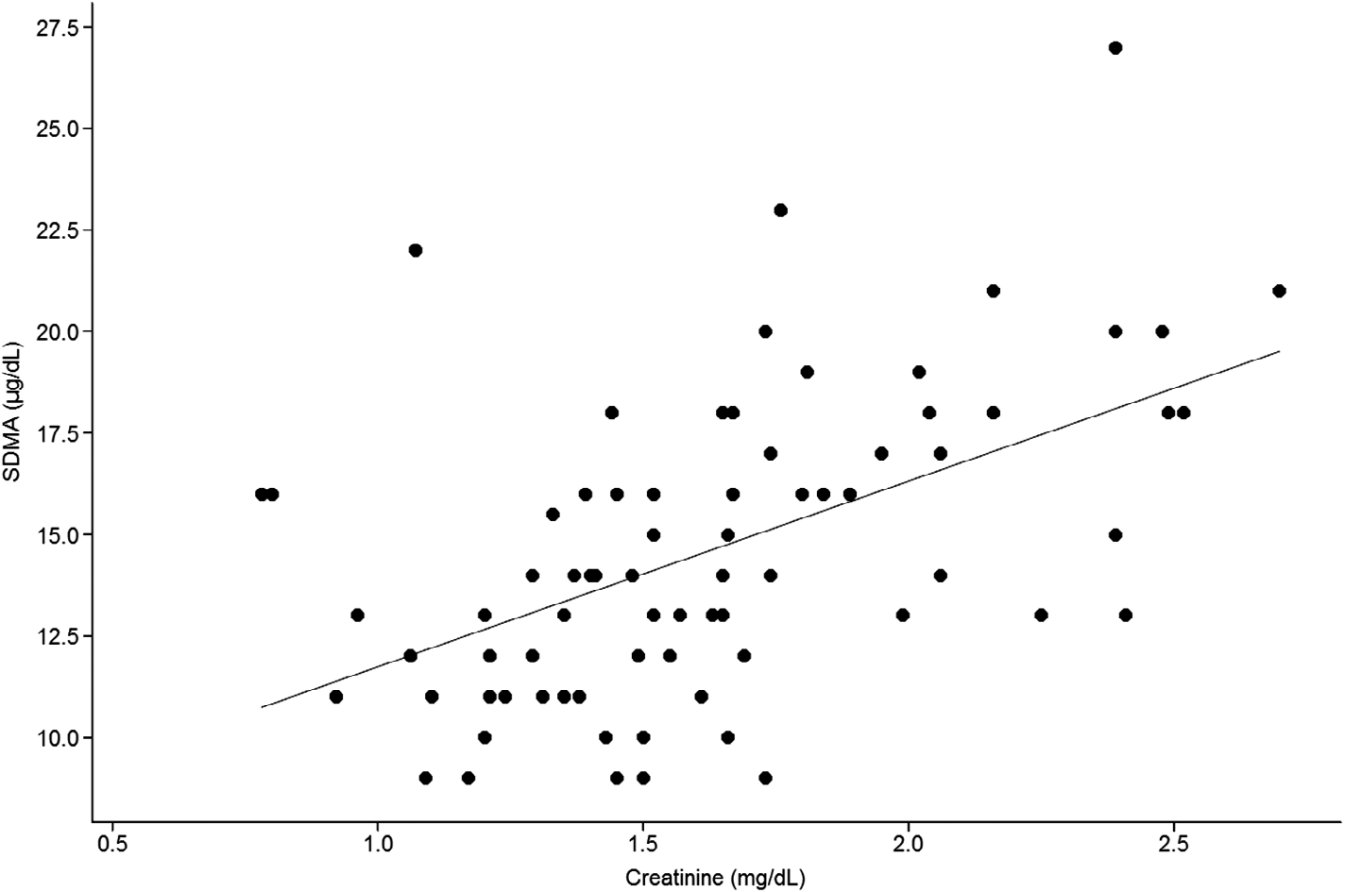
Correlation between serum creatinine and serum symmetric dimethylarginine (SDMA) in 76 cats after radioiodine treatment, excluding the outlier *Note:* Each dot represents 1 cat. The solid line represents the line of best fit

Moderate significant correlation was found between SDMA and creatinine at T1 (*r* = 0.53; 95% CI, 0.33, 0.68; *P* < .001) when the entire sample was analyzed. One cat was identified as an outlier, having a serum SDMA concentration of 118 μg/dL, which was approximately 8 SDs above the mean SDMA concentration. This cat also was classified as an outlier at T0. The cat's SDMA concentration had increased further at T1, whereas it remained classified as possible IRIS stage 1 CKD based on serum creatinine concentration and USG. This cat had a serum TT4 concentration within the reference interval at T1. When this outlier cat was removed from the data, a moderate correlation between serum SDMA and creatinine concentration remained (*r* = 0.54; 95% CI, 0.34, 0.69; *P* < .001; Figure 2). The correlation between SDMA and creatinine also was assessed in the sample subgroups of cats with USG <1.035 and ≥1.035 at T0 and T1, respectively, and results were similar to those obtained for the entire sample. That is, in both subgroups, no significant correlation was found between SDMA and creatinine at T0 but a moderate correlation was found at T1 (data not shown). Multivariable analysis failed to identify any significant influence of extrarenal factors such as BCS and MCS on SDMA and creatinine at both T0 and T1. Similarly, no significant influence from the use of sedation at T0 was identified on the outcome of SDMA and creatinine. This finding could not be formally assessed at T1 given the limited number of cats receiving sedation at this time (Supporting Information Tables S4 and S5).

### 3.5 Relationships among serum SDMA, creatinine, and TT4 concentrations

The relationship between SDMA and TT4 and between creatinine and TT4 at T0 and T1 was assessed in 77 and 80 cats, respectively.

No significant correlation was found between serum SDMA and TT4 concentrations at T0 (*r* = −0.13; 95% CI, −0.34, 0.10; *P* = .27) and T1 (*r* = −0.02; 95% CI, −0.24, 0.20; *P* = .87). Two outliers were identified in the SDMA data at T0 and 1 outlier was identified at T1, as previously described. The correlation between SDMA and TT4 did not change significantly when these outliers were excluded at T0 (*r* = −0.09, 95% CI, −0.31, 0.15; *P* = .44) and T1 (*r* = −0.03; 95% CI, −0.25, 0.20; *P* = .83).

A moderate and significant correlation was found between serum creatinine and TT4 concentrations before radioiodine treatment (*r* = −0.38; 95% CI, −0.55, −0.15; *P =* .001); this relationship weakened after treatment (*r* = −0.20; 95% CI, −0.41, 0.02; *P* = .07).

Two cats that remained hyperthyroid at T1 were identified as outliers because both were > 5 SDs above the mean TT4 concentration. Upon their exclusion, the correlation between SDMA and TT4 did not change significantly (*r* = −0.02; 95% CI, −0.25, 0.21; *P* = .85), and the relationship between and creatinine and TT4 further weakened (*r* = −0.16; 95% CI, −0.37, 0.07; *P =* .16).

## 4 DISCUSSION

We prospectively assessed serum SDMA, creatinine and TT4 concentrations in a sample population of 80 Australian cats before and after treatment of hyperthyroidism with a PO fixed dose of radioiodine. Consistent with the study hypothesis and recently published results,^18^ mean SDMA concentration increased significantly as hyperthyroidism resolved. This is presumably because serum SDMA concentration correlates with GFR^16^, ^29^ and an increase in serum SDMA concentration is expected as the effects of hyperthyroidism on renal perfusion resolve.^5^, ^30^, ^31^ However, serum SDMA concentration did not increase in 21 (28%) of 75 cats, 5 of which had serum TT4 concentrations below the reference interval at T1. In these cats, the concurrent decrease in serum SDMA concentration is particularly surprising because several of these cats may have been hypothyroid, and GFR is expected to decrease in approximately half of cats with iatrogenic hypothyroidism.^32^, ^33^

Similar to previous studies, SDMA and creatinine were not correlated before radioiodine treatment, with only moderate correlation seen after treatment.^18^, ^19^ At T0, 76% of cats with USG <1.035 had agreement on possible IRIS stage 1 CKD based on both serum SDMA and creatinine concentration. The discordances in the remaining 4 cats were because of serum SDMA concentration classifying cats into higher IRIS stages compared with the classification based on serum creatinine concentration, suggesting a higher sensitivity of SDMA for detecting renal dysfunction.^18^ However, at T1, serum SDMA concentration classified cats with USG < 1.035 into both higher and lower IRIS stages than did serum creatinine concentration. This finding is inconsistent with the hypothesis that serum SDMA concentrations would always be increased in cats with suspected renal dysfunction (based on USG <1.035 with concurrently increased serum creatinine concentration) after radioiodine treatment. In contrast to SDMA, serum creatinine concentration increased in all but 2 cats at T1 compared with T0. In support of our findings, another study did not find a significant correlation between serum SDMA concentration and GFR before or after radioiodine treatment in a small group of hyperthyroid cats, whereas serum creatinine concentration and GFR correlated moderately and significantly.^19^ Taken together, these results suggest that SDMA lacks specificity for detecting renal dysfunction in hyperthyroid cats. This findings contrasts findings of a previous study that reported a specificity of 97.7% for pretreatment serum SDMA concentration in detecting masked CKD in untreated hyperthyroid cats.^18^

Thyroid status, renal function and SDMA appear to have a complex interplay. The correlation between serum TT4 and SDMA concentrations were very weak at T0 and T1, a finding supported by other recently published studies.^18^, ^19^ As in a previous study,^19^ we propose that extrarenal factors influence SDMA in hyperthyroid cats. Sedation, BCS and MCS did not appear to influence the trend in serum SDMA or creatinine concentrations at either time point when assessed using multivariable analysis. Dual energy X‐ray absorptiometry analysis assesses body composition more accurately than does subjective scoring systems, and could be applied in the future to assess relationships among body composition, SDMA and creatinine.^34^, ^35^, ^36^ Sedation was used infrequently in the study cats, limiting the analysis of its effects on serum SDMA and creatinine concentrations. Various sedation protocols had no significant effect on GFR in another small study.^37^ Nonetheless, appropriately sized studies are required to confirm the relationships among sedation, GFR, SDMA and creatinine. Prerenal azotemia also may affect serum creatinine and SDMA concentrations. However, dehydration was not evident during physical examination or evaluation of total protein concentrations. In particular, 18 (86%) of 21 cats with decreasing serum SDMA concentration between T0 and T1 had an increase in total protein concentration during that time. A higher total protein concentration at T1 may reflect subclinical dehydration, or the resolution of subclinical gastrointestinal dysfunction associated with hyperthyroidism.^38^, ^39^, ^40^

Apart from the abovementioned extrarenal factors, another study proposed that serum SDMA concentrations in hyperthyroid cats may be affected by alterations in protein metabolism and potential alterations in hepatic clearance of SDMA.^19^ Upregulation of SDMA production also is a possible reason, but so far, it has been reported only in growing animals.^41^ Lastly, genetic variation could contribute to interindividual variability in SDMA metabolism. In people, a genetic variant of the AGXT2 gene has been linked to enhanced SDMA metabolism in vitro^42^; this variation remains to be assessed in cats. Extrarenal factors also may explain the extremely high serum SDMA concentrations in the 2 outliers of our study. In 1 of these cats, serum SDMA concentration increased further at T1, whereas the serum SDMA concentration normalized in the other cat. Both cats were considered to have possible IRIS stage 1 CKD based on their serum creatinine concentration and USG at T0, and had no clinical evidence of dehydration or hypovolemia at either T0 or T1. A separate serum sample from the cat with the extremely high SDMA concentration of 118 μg/dL at T1 was sent to the reference laboratory and yielded corroborating results. So far, such extreme SDMA results have not been reported in the human or veterinary literature. Further investigations, such as diagnostic imaging, were not performed and it cannot be excluded that the cat with increasing serum SDMA concentration had occult kidney disease or nephrolithiasis.^43^

Our study sample population is comparable to that of recent studies assessing renal parameters before and after radioiodine treatment in cats^18^, ^19^, ^44^; namely, geriatric, predominantly domestic breed, hyperthyroid cats. Female neutered cats were slightly overrepresented in our study, but it also has been observed in other studies.^18^, ^19^ The proportions of cats with apparent decreased renal function at T0 and T1 are similar to existing reports in the literature,^1^, ^2^, ^3^, ^7^, ^45^ and as previously reported, renal azotemia was more common in cats with serum TT4 concentrations below the lower limit of the reference interval after radioiodine treatment than in those with serum TT4 concentrations within the reference interval.^18^, ^44^

The chosen follow‐up time after radioiodine treatment was approximately 3 months. Although the expected recovery time for atrophic thyroid tissue after resolution of hyperthyroidism is 1 to 3 months in most cats,^46^, ^47^, ^48^ recent evidence indicates a large proportion of cats could develop overt hypothyroidism as late as 6 to 12 months after radioiodine treatment.^32^ This could further influence renal function such that results of our study may not accurately reflect long‐term thyroid function in cats. However, most changes in renal function related to resolution of hyperthyroidism occur in the first month after radioiodine treatment, with no further significant change in GFR between 1 and 6 months after treatment.^5^ Furthermore, up to 30% of previously healthy geriatric cats will develop CKD over the course of 12 months.^49^ Such an occurrence could confound interpretation of results if follow‐up periods after radioiodine treatment increase beyond 3 months.

Our study had some limitations. First, we were unable to measure GFR concurrently with other variables of renal and thyroid function. Second, the study concentrated solely on measurement of serum TT4 concentration; a complete assessment of thyroid status was not performed. Hence, we could not define subpopulations with iatrogenic or subclinical hypothyroidism. Furthermore, the precise serum TT4 concentration was not known for cats with results >15 μg/dL (>193.0 nmol/L) at T0 and <0.5 μd/dL (<6.4 nmol/L) and at T1. This may have confounded assessment of the relationship between renal variables and serum TT4 concentration at both time points. Third, we selected IRIS cut‐offs for serum SDMA and creatinine concentration in CKD staging in preference to the laboratory upper reference intervals. This influenced the number of cats categorized as suspected IRIS stage 2 (azotemic) CKD. This approach was taken to enable comparison of results with future studies and for standardized gradation of severity. Lastly, it is a limitation that CKD staging before or after radioiodine treatment was based on a single measurement of serum creatinine and SDMA concentration. High interindividual variability occurs in serum SDMA and creatinine concentrations and sequential measurements are preferred for confirmation and staging of CKD.^8^, ^50^ It is possible that the single measurements performed here misclassified and confounded comparison of CKD staging by serum SDMA or creatinine concentration. Accurate CKD staging also may have been limited by suspicion of CKD diagnosis based solely on a single USG of <1.035. Urine specific gravity can be influenced by extrarenal factors such as hydration status and thyrotoxic effects of hyperthyroidism resulting in loss of ability to concentrate urine or primary polydipsia.^8^, ^40^ Further evaluation of SDMA in cats with hyperthyroidism calls for larger‐scale, prospective longitudinal studies with concurrent GFR measurements and full assessment of thyroid function status before and after treatment.

In summary, in our prospective cohort study, which assessed serum creatinine, SDMA and TT4 concentrations in hyperthyroid cats before and after treatment with a fixed PO dose of radioiodine (approximately 3.6 mCi; 131.6 MBq) we found that SDMA concentrations increased inconsistently after treatment, suggesting that extrarenal factors affect serum SDMA concentrations in hyperthyroid cats. Further study is needed to evaluate the relationships among SDMA, renal status, and thyroid status in cats. Meanwhile, serum SDMA and creatinine concentrations cannot be substituted for each other, and serum SDMA concentrations in hyperthyroid cats should not be interpreted in isolation, because they may not adequately reflect renal function in these cats.

## CONFLICT OF INTEREST DECLARATION

Authors declare no conflict of interest.

## OFF‐LABEL ANTIMICROBIAL DECLARATION

Authors declare no off‐label use of antimicrobials.

## INSTITUTIONAL ANIMAL CARE AND USE COMMITTEE (IACUC) OR OTHER APPROVAL DECLARATION

This study was approved by and conducted in accordance with the University of Melbourne Animal Ethics Committee; AEC application ID 1613858. Written informed consent was obtained from the owners of all animals described in this work for the procedures undertaken.

## HUMAN ETHICS APPROVAL DECLARATION

Authors declare human ethics approval was not needed for this study.

## Supporting information


**Data S1** Supporting Information
