# Supplementary material for: Assessment of serum symmetric dimethylarginine and creatinine concentrations in hyperthyroid cats before and after a fixed dose of orally administered radioiodine
Source: J Vet Intern Med. 2020 Jun 7;34(4):1423–31. doi: 10.1111/jvim.15831 (PMC7379014; doi:10.1111/jvim.15831)
Supplement: Supplementary file 1 — Data S1 Supporting Information [file JVIM-34-1423-s001.pdf]

Supplementary Table 1. Mean body weight, body condition score, muscle condition score and systolic blood pressure in 80 hyperthyroid cats before (T0) and after (T1) radioiodine treatment

	T0	T1	Estimated difference; T1-T0 (95% confidence interval)	*p*-value
	**Mean (SD)**	**Mean (SD)**		**Paired t-test**
Body weight (kg)	4.2 (1.1)	4.7 (1.2)	0.5 (0.4, 0.6)	<0.001
Blood pressure (mmHg)	178.9 (29.45)	160.8 (32.43)	-18.1 (-27.7, -8.6)	<0.001
	**Median (IQR)**	**Median (IQR)**		**Wilcoxon signed-rank test**
Body condition score (out of 9)	4.0 (3-5)	5.0 (4-5)	1 (0.5,1)	<0.001
Muscle condition score (out of 4)	3 (2-3)	4 (3-4)	1 (0.5,1)	<0.001

Legend: T0, before treatment; T1, after treatment

Supplementary Table 2. Clinicopathologic data before (T0) and after (T1) radioiodine treatment in 66 cats with a total thyroxine concentration within the reference interval after treatment.

	T0		T1		Estimated mean difference; T1-T0 (95% confidence interval)	*p*-value*
	n	Mean (SD)	n	Mean (SD)		
PCV (%)	66	36.4 (4.3)	66	34.1 (0.0)	-2.3 (-3.3, -1.3)	<0.001
TP (g/dL)	66	68.5 (5.6)	66	73.9 (5.0)	5.4 (4.0, 6.9)	<0.001
Serum TT4 (µg/dL)	66	10.7 (4.3)	66	1.6 (0.5)	-9.11 (-10.2, -8.0)	<0.001
Serum Creatinine (mg/dL)	66	0.94 (0.34)	66	1.6 (0.4)	0.65 (0.6, 0.8)	<0.001
Serum urea (mg/dL)	66	8.5 (2.6)	66	10.2 (2.6)	1.7 (1.2, 2.2)	<0.001
Serum SDMA (µg/dL)	63	12.7 (5.9)	63	16.5 (13.5)	3.8 (1.4, 6.2)	0.002
Urine specific gravity	62	1.041 (0.010)	62	1.033 (0.014)	-0.007 (-0.010, -0.004)	<0.001

Legend: *Paired t-test; T0, before treatment; T1, after treatment; TP, total protein; TT4, total thyroxine; SDMA, symmetric dimethylarginine

Supplementary Table 3. Clinicopathologic data before and after radioiodine treatment in 12 cats with a total thyroxine concentration below the reference interval after treatment

	Before treatment		After treatment		Estimated mean difference; T1-T0 (95% confidence interval)	*p*-value*
	n	Mean (SD)	n	Mean (SD)		
PCV (%)	12	36.6 (6.9)	12	31.7 (4.9)	-4.9 (-8.0, -1.8,)	0.005
TP (g/dL)	12	70.9 (5.3)	12	79.0 (5.2)	8.1 (4.3, 11.9)	0.001
Serum TT4 (µg/dL)	12	9.3 (5.5)	12	0.55 (0.1)	-8.8 (-12.3, -5.3)	<0.001
Serum Creatinine (mg/dL)	12	1.0 (0.3)	12	1.8 (0.5)	0.8 (0.6, 1.1)	<0.001
Serum urea (mg/dL)	12	23.8 (6.7)	12	35.9 (11.8)	12.0 (5.6, 18.5)	<0.001
Serum SDMA (µg/dL)	10	16.0 (13.10)	10	12.3 (3.9)	-3.7 (-12.6, 5.1)	0.36
Urine specific gravity	12	1.040 (0.009)	12	1.033 (0.013)	-0.007 (-0.013, 0)	0.04

Legend: *Paired t-test; T0 before treatment; T1, after treatment; TP, total protein; TT4, total thyroxine; SDMA, symmetric dimethylarginine

Supplementary Table 4. Multivariable analysis using a general linear model to assess the effect of extra-renal variables on SDMA at before (T0) and after (T1) radioiodine treatment.

Extra-renal variables	Time	df	F	Direction of difference	Difference of means Estimate	95% CI	*P*-value
Body condition score	T0	1, 62	0.24	9-1	-0.0076	-0.0385, 0.0233	0.626
	T1	1, 71	0.03	9-1	-0.00089	-0.01056, 0.00878	0.854
Muscle condition score	T0	1, 62	1.56	4-0	-0.0266	-0.0692, 0.0160	0.216
	T1	1, 71	0.20	4-0	0.00394	-0.01354, 0.02142	0.655
Sedation	T0	1, 62	2.10	Yes-No	0.0218	-0.0082, 0.0518	0.152

Legend: T0, before treatment; T1, after treatment

Supplementary Table 5. Multivariable analysis using a general linear model to assess the effect of extra-renal variables on creatinine at before (T0) and after (T1) radioiodine treatment.

Extra-renal variables	Time	df	F	Direction of difference	Difference of means		*P*-value
					Estimate	95% CI	
Body condition score	T0	1, 65	0.11	9-1	2.73	-13.62, 19.07	0.740
	T1	1, 62	0.05	9-1	0.014	-0.113, 0.141	0.822
Muscle condition score	T0	1, 65	0.03	4-0	-2.00	-23.80, 19.80	0.858
	T1	1, 62	0.20	4-0	0.052	-0.179, 0.284	0.653
Sedation	T0	1, 65	0.19	Yes-No	-3.40	-19.17, 12.37	0.668

Legend: T0, before treatment; T1, after treatment

Supplementary Figure 1. Serum creatinine of 80 cats before (T0) and after (T1) radioiodine treatment.

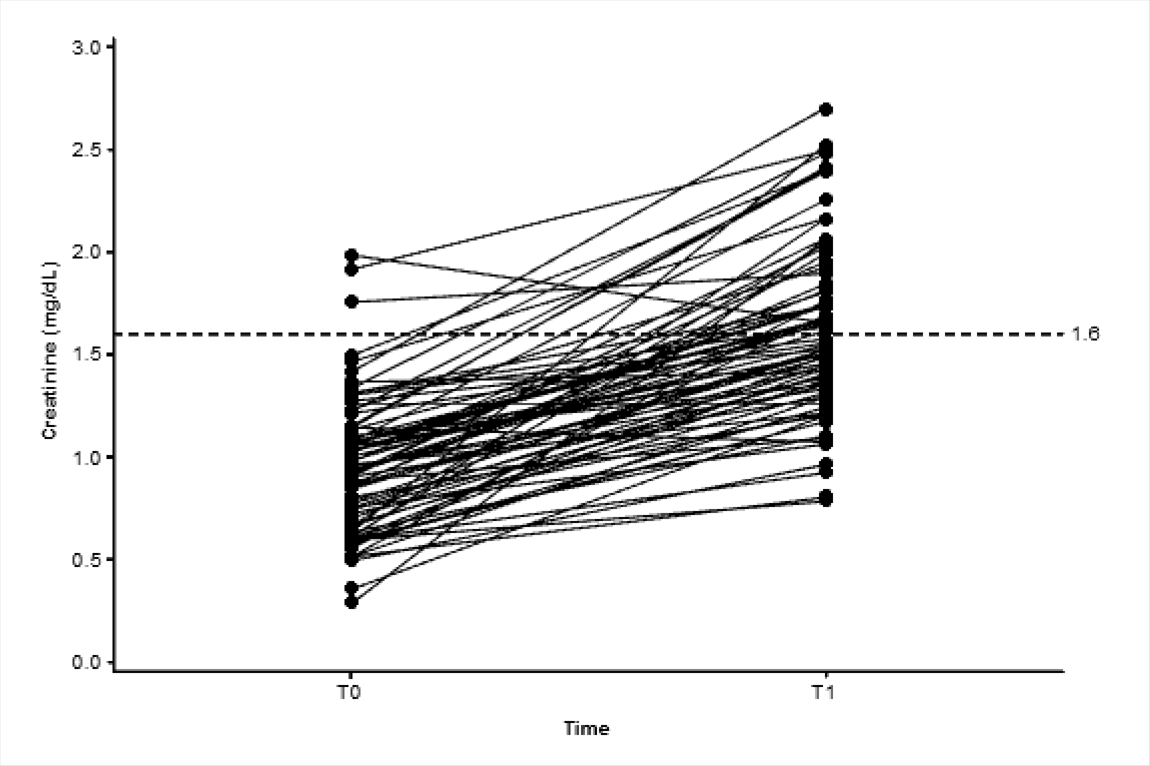

Legend: T0, before treatment; T1, after treatment. The dotted line represents the serum creatinine cut-off value for renal azotemia according to International Renal Interest Society chronic kidney disease staging guidelines.

Supplementary Figure 2. Urine specific gravity in 76 Cats before (T0) and after (T1) radioiodine treatment.

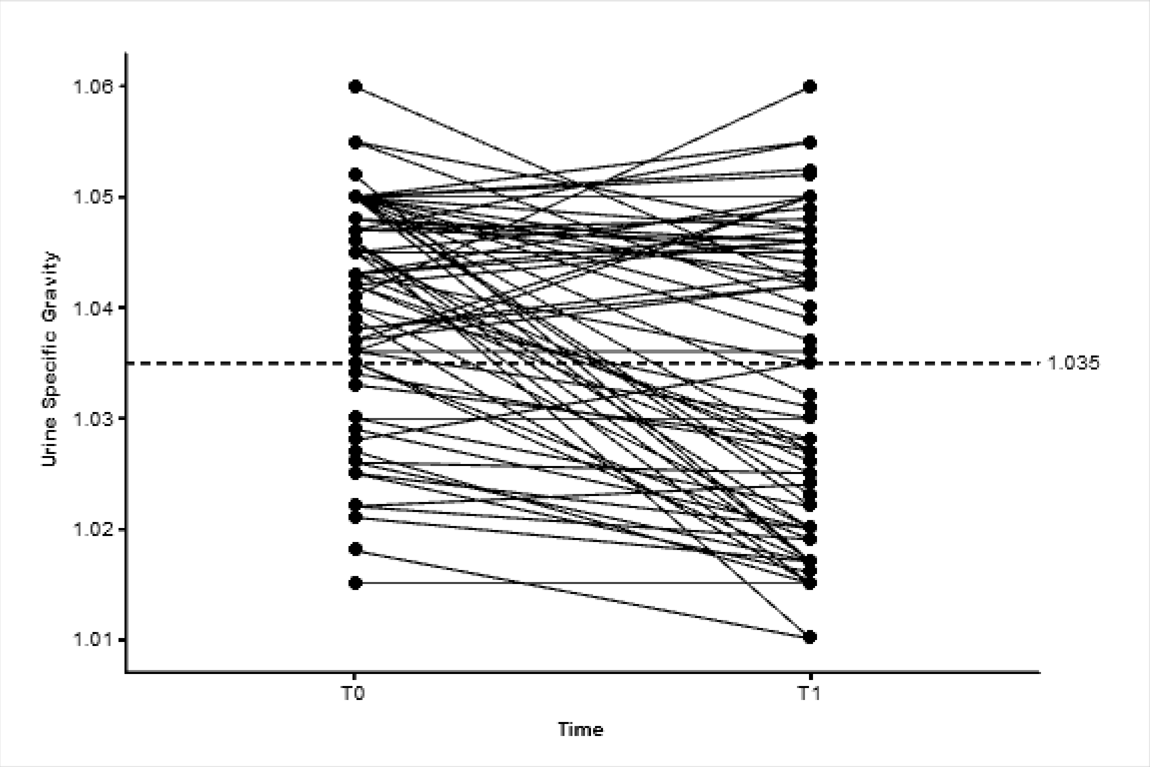

Legend: T0, before treatment; T1, after treatment. The dotted line represents the lower cut-off for adequate urine concentration according to International Renal Interest Society chronic kidney disease staging guidelines.
